# Quantifying mRNA and MicroRNA with qPCR in Cervical Carcinogenesis: A Validation of Reference Genes to Ensure Accurate Data

**DOI:** 10.1371/journal.pone.0111021

**Published:** 2014-11-03

**Authors:** Maria da Conceição Gomes Leitão, Eliane Campos Coimbra, Rita de Cássia Pereira de Lima, Mariléa de Lima Guimarães, Sandra de Andrade Heráclio, Jacinto da Costa Silva Neto, Antonio Carlos de Freitas

**Affiliations:** 1 Laboratory of Molecular Studies and Experimental Therapy (LEMTE), Department of Genetics, Center for Biological Sciences, Federal University of Pernambuco, Pernambuco, Brazil; 2 Clinical Hospital of Federal University of Pernambuco, Pernambuco, Brazil; 3 Institute of Integral Medicine Prof. Fernando Figueira, Pernambuco, Brazil; 4 Molecular and Cytological Research Laboratory, Department of Histology, Federal University of Pernambuco, Pernambuco, Brazil; University of Houston, United States of America

## Abstract

A number of recent studies have catalogued global gene expression patterns in a panel of normal, tumoral cervical tissues so that potential biomarkers can be identified. The qPCR has been one of the most widely used technologies for detecting these potential biomarkers. However, few studies have investigated a correct strategy for the normalization of data in qPCR assays for cervical tissues. The aim of this study was to validate reference genes in cervical tissues to ensure accurate quantification of mRNA and miRNA levels in cervical carcinogenesis. For this purpose, some issues for obtaining reliable qPCR data were evaluated such as the following: geNorm analysis with a set of samples which meet all of the cervical tissue conditions (Normal + CIN1 + CIN2 + CIN3 + Cancer); the use of individual Ct values versus pooled Ct values; and the use of a single (or multiple) reference genes to quantify mRNA and miRNA expression levels. Two different data sets were put on the geNorm to assess the expression stability of the candidate reference genes: the first dataset comprised the quantities of the individual Ct values; and the second dataset comprised the quantities of the pooled Ct values. Moreover, in this study, all the candidate reference genes were analyzed as a single “normalizer”. The normalization strategies were assessed by measuring p16^INK4a^ and miR-203 transcripts in qPCR assays. We found that the use of pooled Ct values, can lead to a misinterpretation of the results, which suggests that the maintenance of inter-individual variability is a key factor in ensuring the reliability of the qPCR data. In addition, it should be stressed that a proper validation of the suitability of the reference genes is required for each experimental setting, since the indiscriminate use of a reference gene can also lead to discrepant results.

## Introduction

Cervical cancer is one of the most common cancers affecting women worldwide and is linked to human papillomavirus (HPV) infection [1], [2]. This type of cancer is preceded by preventable precancerous lesions; however, conventional screening tests lack both sensitivity (in the Pap test) and specificity (in the HPV test) [3]–[6]. Hence, there is an urgent need for new effective biomarkers to improve the triage tests and determine how affected women can be treated in an appropriate way [7].

Some studies have catalogued global gene expression patterns in a panel of normal, tumoral cervical tissues so that potential biomarkers can be identified [8], [9]. The real-time quantitative PCR (qPCR) has been one of the most widely used technologies for detecting these potential biomarkers. However, reliable results can only be achieved with this technology by evaluating some crucial parameters [10], [11] such as the validation of reference genes, which must be as stable as possible in the investigated samples [12], [13]. Additionally, some studies have included a pool of samples in the qPCR assays [14]–[17]. This strategy is usually employed to reduce biological variability and also to reduce the costs of the experiments. However searching for variations in gene expression should take account of the endogenous variations of the biological individuals to avoid an erroneous interpretation of the data [18].

Until now, few studies have investigated a correct strategy for data normalization in qPCR assays for cervical tissues; one of them recommended the use of reference genes for mRNA expression studies [19], and another for microRNA (miRNA) expression studies [20]. Nevertheless, several research groups have stressed the importance of evaluating normalization targets as has been demonstrated in the way the results varied in accordance with the choice of reference gene in both the mRNA [21] and miRNA [22] qPCR assays. Additionally, even though it has been well established that the use of a single or unvalidated reference gene is not suitable to obtain reliable qPCR data [12], studies in cervical cancer continue to use the most well-known reference genes such as GAPDH and RNU-6, as single reference gene to measure mRNA and miRNA expression levels, respectively [23]–[27].

In the light of this, the aim of this study was to validate reference genes in cervical tissues to ensure accurate quantification of mRNA and miRNA levels in cervical carcinogenesis. For this purpose, some issues for obtaining reliable qPCR data were evaluated such as the following: geNorm analysis with a set of samples which meet all of the cervical tissue conditions (Normal + CIN1 + CIN2 + CIN3 + Cancer); the use of individual samples (or individual Ct values) versus a pool of samples (or pooled Ct values); and the use of a single (or multiple) reference genes to quantify mRNA and miRNA expression levels.

## Materials and Methods

### Ethics statement

This study was approved by the “Research Ethics Committee of the Federal University of Pernambuco”, Brazil, (number: 03606212.7.0000.5208) and also by the Institutional Review Board of the Clinical Hospital of UFPE, and the Prof. Fernando Figueira Institute of Integral Medicine - IMIP. All the patients signed a written consent form prior to the collection of the samples.

### Patients and samples

The experiments were planned and carried out in accordance with the MIQE guidelines [10]. The biopsies of patients were collected at the Clinical Hospital of UFPE and Institute of Integral Medicine Prof. Fernando Figueira Institute of Integral medicine (IMIP). Biopsies were obtained from women undergoing colposcopy, with different degrees of cervical intraepithelial neoplasia-CIN (CIN 1, 2, 3), and cancer (Ca). Normal cervical tissue samples (negative for neoplasia) were included as controls. Written consent forms were obtained from all the patients, prior to the sample collection. Women with the Human Immunodeficiency Virus (HIV) and/or during pregnancy were excluded from this study. Fresh cervical biopsies were immediately preserved in RNAlater (Qiagen) and stored at −80°C. The biopsies were used in their entirety. HPV detection in samples was performed by PCR [28], after extraction and purification of total DNA with Trizol (Invitrogen) and DNeasy Blood & Tissue Kit (Qiagen), respectively. A total of 65 samples were used that consisted of five groups: CIN1 (12), CIN2 (6), CIN3 (14), cancer (14) and normal cervical tissue (19). All the samples from cancer and CIN were found to be positive for HPV, and all the normal cervical tissue samples were found to be HPV negative.

### Isolation of total RNA and cDNA synthesis

The preserved samples (25–100 mg) were ground while still nitrogen-frozen and homogenized with 1 ml of Trizol (Invitrogen) for isolation of total RNA (including miRNAs and mRNAs), in accordance with the manufacturer's instructions. Total RNA was purified in a subsequent stage by means of the miRNA Absolutely RNA Kit (Agilent Technologies). The quantity and purity of total RNA were estimated by NanoDrop 2000 Spectrophotometer (ThermoScientific), and the criterion for the inclusion of the RNA samples was 260/280 (1.8–2.1). The RNA integrity was assessed by 1% agarose gel electrophoresis, through visualization of intact rRNA subunits (28 S and 18 S).

cDNA was synthesized from 1µg of total RNA using the miScript II RT kit (Qiagen) in a 20 µl reaction volume. The cDNA generated with the aid of the miScript II RT Kit was used as a template for quantification of miRNA and mRNA. An RT-minus negative control reaction with all the components for the RT reaction (except the Reverse Transcriptase enzyme) was carried out for each sample to control genomic DNA contamination.

### Selection of gene sequences and primer design

Four protein coding genes (mRNA genes) were selected for expression analyses (*GAPDH*, *ACTB*, *EEF1A1* and *RPLPO*) based on previous qPCR studies in cervical cancer [19], [23], [29], [30]. The characteristics of each gene, such as the accession number, genomic location, function, and amplicon size are summarized in Table 1. Primers were designed on the basis of the sequence data obtained from GenBank (http://www.ncbi.nlm.nih.gov/) using the CLCBioMain Workbench 5.7.1 software (Table 2).

**Table 1:** Characteristics of the mRNA genes selected for the stability analysis.

Gene	Acession Number (GenBank)	Gene Name	Genomic Localization	Function
ACTB	001101	Beta-Actin	7p15–p12	Structural cytoskeletal protein
GAPDH	002046	Glyceraldehyde-3-phosphate dehydrogenase	12p13	Oxidoreductase-glycolysis and gluconeogenesis
EEF1A1	001402	Eukaryotic translation elongation factor 1 alpha 1	6q14.1	Elongation factor of translation in eukaryotes
RPLPO	002046	Large ribosomal protein	12q24.2	Ribosomal protein

**Table 2:** Primer pair sequences for amplification of mRNA genes.

Gene	Primer sequence	Amplicon Size (bp)
ACTB	F: TCGAGC AAGAGATGGCCAC	132
	R: GGAAGGAAGGCTGGA AGAGT	
GAPDH	F: GAAGGCTGGGGCTCATTTG	91
	R: TAAGCAGTTGGTGGTGCAGG	
EEF1A1	F: GTTGCGGTGGGTGTCATC A	123
	R: GAGTGGGGTGGCAGGTAT T	
RPLPO	F: GCTGCTGCCCGTGCTGGTG	130
	R: TGGTGCCCCTGGAGATTTTAGTGG	

In the case of the analysis involving miRNA expression studies, three non-protein coding genes (npcRNA genes) were selected for the evaluation of stability: *RNU6-2*, *miR-191* and *miR-23a*. These genes are small npcRNAs and correspond to the family of snoRNAs and microRNAs, respectively, which are commonly used as reference genes, not only in cervical tissues, but also in other types of tissues [15], [20], [22], [31]. The primers were purchased from miScript primer assay (Qiagen) and from miScript PCR Starter Kit (Qiagen), which contains the miScript Universal Primer. The characteristics of each npcRNA gene are summarized in Table 3.

**Table 3:** Characteristics of the npcRNA genes selected for the stability analysis.

Gene	Acession Number (GenBank)	Genomic Localization	RNA species	Function
miR-191	406966	3p21.31	miRNA	Regulation of processes such as apoptosis and cell cycle
miR-23a	407010	19p13.13	miRNA	Involved in myoblasts proliferation and differentiation
RNU6-2	26826	10p13	snoRNA	Involved in chemical modifications of RNAs

### Real-time quantitative polymerase chain reaction (qPCR)

The Rotor Gene 6000 thermocycler (Qiagen) was used to run the qPCR reactions. The reactions were in duplicate and the final volume for each reaction was 20 µl, containing 10 µl of 2X QuantiTect SYBR Green PCR kit (Qiagen), 1 µl of forward primer, 1 µl of reverse primer, 6 µl of RNAse-free water and 2 µl of cDNA. The final concentration of each primer in the PCR reaction was 0.5 µM. The final concentration of cDNA was 20 ng per qPCR reaction for the mRNA measurement, and 2 ng of cDNA per qPCR reaction for microRNA measurement. Negative controls without cDNA for each primer pair were added to detect contamination. Negative controls of cDNA synthesis (not submitted to the reverse transcriptase action) were also added to detect possible contamination with genomic DNA. The reaction conditions for the quantification of mRNA were as follows: 15 min at 95°C (initial activation of HotStarTaq DNA Polymerase), followed by 30 cycles of 95°C for 25 s, 60°C for 25 s, and 72°C for 25 s, with a final extension at 72°C for 2 min. For miRNA qPCR, the conditions were: 15 min for 95°C (initial activation of HotStartTaq DNA Polymerase), followed by 40 cycles of 94°C for 15 s, 55°C for 30 s and 70°C for 30 s.

The amplification efficiency for each primer pair was determined by a qPCR assay using triplicates of a 10-fold dilution series (1:10, 1:100, 1:1000, 1:10.000, 1:100.000) of normal cervical tissue cDNA as a template. The mean Ct values for each serial dilution were plotted against the logarithm of the cDNA dilution factor. The amplification efficiency for each primer pair was calculated by standard curve methods using the Efficiency  = (10^(−1/slope)^-1)×100 formula. The melting curve was obtained to confirm the specificity of the primers.

### Analysis of gene expression stability

The software program used to calculate the expression stability of reference candidate genes was geNorm [12]. The geNorm calculates the average expression stability value (M) with a standard deviation between the logarithmically transformed expression rates. This M value is the average pairwise variation of one particular gene compared to all the other tested genes. This program recommends using an M below the threshold of 1.5 to identify the reference genes. The geNorm also estimates the pair-wise variation value (Vn/n +1), by allowing the identification of the optimal number of reference genes to be used. Pair-wise variation values with a threshold ≤0.15 are considered sufficient for normalization, although this limit should not be seen as a very narrow cut-off point. Thus, the geNorm indicates the number of genes necessary for normalization through normalization factors (NF_n_) or providing the geometric means of combining the most stable reference genes: the two most stable genes (NF_2_), the three most stable genes (NF_3_), etc.

Two different data sets were put on the geNorm to assess the expression stability of the candidate reference genes: the first dataset comprised quantities from individual Ct values; and the second dataset comprised quantities from pooled Ct values. In the first geNorm analysis, the large number Ct values from independent replicates in each cervical tissue condition were taken into account. Thus, assuming that 65 samples - CIN1 (12), CIN2 (6), CIN3 (14), cancer (14), normal (19) - were used in the duplicate for each qPCR assay, there were 130 Ct values per gene. In the second geNorm analysis, account was taken of the arithmetic mean of Ct values per gene per cervical tissue condition. Briefly, the sum of the individual Ct values (including biological replicates and technical repeats) was divided by the total number of samples. For instance, five average values were obtained per each candidate gene from the cervical tissue conditions: 24 Ct values from CIN 1 (from 12 biological replicates ×2 PCR repeats), 12 Ct values from CIN 2 (6×2), 28 Ct values from CIN 3 (14×2), 28 Ct values from cancer (14×2), and 38 Ct values from normal (19×2). It should be noted that these two types of data (individual Ct values and pooled Ct values) were applied to the mRNA genes (*GAPDH*, *ACTB*, *EEF1A1*, *RPLPO*) and npcRNA genes (*RNU-6*, *miR-23a*, *miR-191*), in two independent analyzes conducted by means of geNorm.

### Validation of reference genes in cervical tissues

In order to validate the most stable genes recommended by geNorm as suitable reference genes for normalization of qPCR data in cervical carcinogenesis, two targets were evaluated. Thus, the two most stable genes, the three most stable genes and the two least stable genes were used to normalize the expression levels of the chosen targets (p16^INK4a^ and miR-203) for each sample (in the same batch of cDNA). The p16 primer pair (F_ACATCCCCGATTGAAAGAACC; R_ATGAAAACTA CGAAAGCGGGG) was designed on the basis of the GenBank data (ID: 1029) with the aid of CLCBioMain Workbench 5.7.1 software. The primers for miR-203 amplification was purchased from the miScript primer assay (Qiagen) and the miScript PCR Starter Kit (Qiagen), which contains the miScript Universal Primer. Moreover, with the purpose to demonstrate the effect of using a single reference gene on the target expression, we assessed the relative expression of p16^INK4^ obtained by each of the four single reference genes (GAPDH, ACTB, EEF1A1, RPLPO), and the relative expression of miR-203 obtained by each of the three single reference genes (RNU-6, miR-23a, miR-191). In employing this normalization strategy, the linear scale expression quantities of the reference genes obtained from the individual Ct values, as well as from the pooled Ct values, were directly used to calculate the relative quantification of the targets. The Ct values of each target were not pooled and each biological replicate was kept independent (65 biological samples ×2 PCR repeats  = 130 Ct values per target) since our objective was only to assess the effect of the pooled Ct values for the reference genes.

### Statistical analysis

A statistical analysis was conducted by making use of two kinds of software: R (version 3.1.0) and GraphPad Prism (version 6.0). Before the geNorm analysis the D′Agostino–Pearson normality test was carried out to determine the distribution of the data. A one-way analysis of variance (ANOVA) was employed to compare the relative quantities of the p16^INK4^ and miR-203 targets across all the cervical tissue conditions. The Bonferroni correction was used to correct P values. The P value <0.05 was considered as statistically significant.

## Results

### Determination of RNA quality and qPCR efficiency

The RNA concentrations from cervical tissues were suitable and ranged from 200-3000 ng/µl, depending on the size of the biopsy (25–100 mg). All these RNA samples were checked for purity and integrity. The value of the purity ranged from 1.8 to 2.0, in accordance with the absorbance ratio at 260/280 nm. The integrity was visualized by the presence of intact 28 S and 18 S ribosomal subunits on electrophoresis gel. Thus, all the RNA samples included in this study were reliable and were representative of the evaluated tissues.

The qPCR efficiency was determined for each primer pair by using the slope of a linear regression model (Figure S1). All the PCR primer pairs showed correlation coefficients of R2 = 0.99 and primer efficiency values (E) ranging from 0.99 to 1.00 (Table 4). The specificity of all the primer pairs was confirmed by a single peak in the melting curve (Figure S2).

**Table 4:** PCR efficiency for all primer pairs.

Gene	Slope	Efficiency	R2
ACTB	−3.30056	1.00901	0.99521
GADPH	−3.27585	1.01959	0.99643
RPLPO	−3.31783	1.00171	0.99495
EEF1A1	−3.27014	1.02208	0.99838
p16^INK4a^	−3.27224	1.02117	0.99851
RNU6	−3.32510	0.99868	0.99706
miR-191	−3.29939	1.00949	0.99121
miR-23a	−3.30039	1.00941	0.99501
miR-203	−3.24109	1.02142	0.99873

### Expression ranges of candidate normalizers in cervical tissues

**Figure 1:**
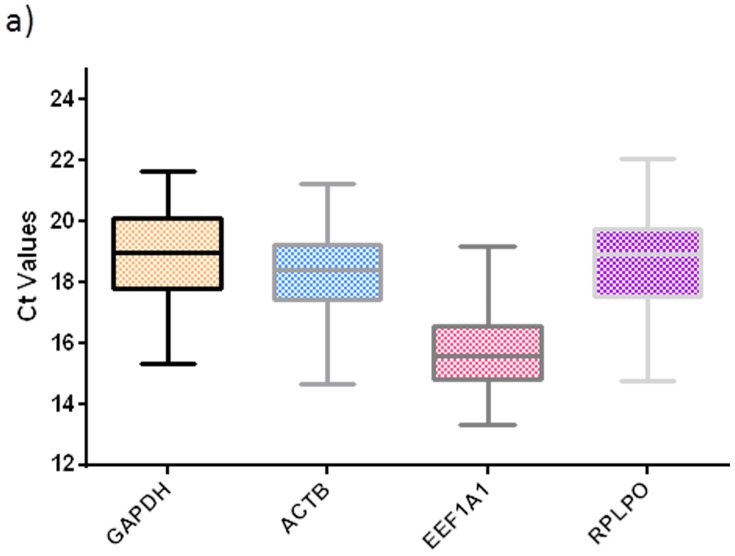
Ct values of the candidate mRNA reference genes in cervical tissues. Boxplots shows interquartile range box, median and range whiskers, from the raw Ct values obtained from the amplification curves. All the genes showed a normal distribution pattern across all the cervical tissue conditions as confirmed by the D′Agostino–Pearson normality test.

In this study we evaluated the expression pattern of the most commonly used reference genes (protein coding genes and non-protein coding genes) for qPCR assays in cancer research. As can be seen in Figure 1, the *GAPDH*, *ACTB*, *EEF1A1* and *RPLPO* showed Ct values between 13 (*EEFA1*) and 22 (*RPLPO*) in all the cervical tissues conditions evaluated. The *EEF1A1* was the most abundant transcript with Ct values ranging from 13.3 to 19.2, and *RPLPO* was the least abundant transcript with Ct values ranging from 20.0 to 22.0. The Ct values of the *GAPDH* and *ACTB* transcripts were similar and ranged from 14.6 to 19.9 for *ACTB*, and 15.3 to 21.6 for *GAPDH* (Figure 1).

**Figure 2:**
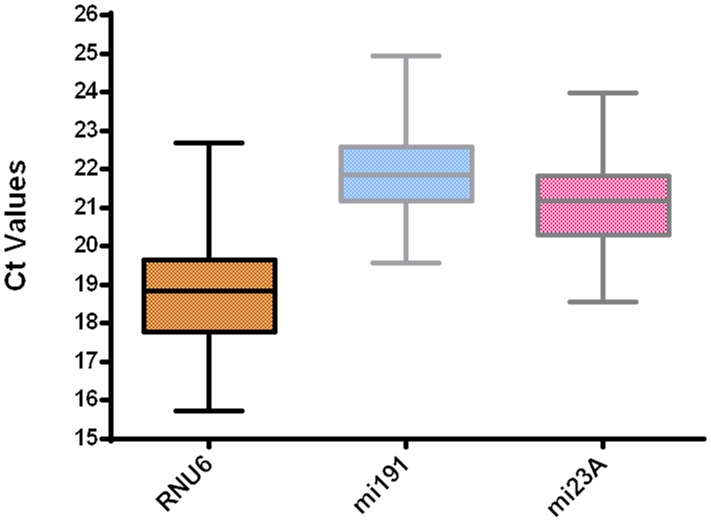
Ct values of candidate npcRNA reference genes in cervical tissues. Boxplots shows interquartile range box, median and range whiskers, from the raw Ct values obtained from the amplification curves. All the npcRNA genes showed a normal distribution pattern across all the cervical tissue conditions as confirmed by the D′Agostino–Pearson normality test.

Figure 2 shows the expression levels of non-protein coding genes (*RNU6*, *miR-191* and *miR-23a*) for all the evaluated cervical tissues. The *RNU6* transcript was the most abundant and had Ct values of 15.7 to 22.6. Inversely, the *miR-191* transcript was the least abundant with Ct values that ranged from 20.0 to 24.9. The *miR-23a* transcript showed Ct values in an intermediate position compared with the other npcRNA genes and ranged from 18.5 to 23.9.

### Determination of the most stable reference genes

The stability ranking and the best combination of genes that could be used as a normalizer, were provided by geNorm after conducting an analysis involving individual Ct values (Table 5) as well as the pooled Ct values (Table 6). All the candidate reference genes showed average expression stability values (M) below the threshold of 1.5 intragroup, as recommended by geNorm. *GAPDH* and *ACTB* were recommended as the most stable genes followed by *EEF1A1* and *RPLPO* from an analysis involving individual Ct values (Table 5). Of all the npcRNA genes, *miR-191* was found to be the most stable, followed by *miR-23a*. *RNU6* was revealed to be the least stable gene (Table 5). The ranking of the mRNA genes (as well as the best combination of genes) was altered in this second analysis which involved pooled Ct values (Table 6). *GAPDH* remained the most stable gene, but *EEFA1* became the second most stable. However, no alteration was observed in the stability ranking of the npcRNA genes (Table 6).

**Table 5:** Ranking and best combination of candidate genes determined from the analysis of individual Ct values, by geNorm.

mRNA genes			npcRNA genes	
M value	Name	Rank	Name	M value
1.28	GAPDH	1	miR-191	1.32
1.28	ACTB	2	miR-23a	1.32
1.31	EEF1A1	3	RNU6	1.44
1.38	RPLPO	4		
GAPDH and ACTB		Best combination	miR-191 and miR-23a	

**Table 6:** Ranking and best combination of candidate genes determined from the analysis of pooled Ct values, by geNorm.

mRNA genes			npcRNA genes	
M value	Name	Rank	Name	M value
0.29	GAPDH	1	miR-191	0.16
0.29	EEF1A1	2	miR-23a	0.16
0.39	ACTB	3	RNU6	0.34
0.74	RPLPO	4		
GAPDH and EEF1A1		Best combination	miR-191 and miR-23a	

### Validation of reference genes for measuring mRNA expression in cervical tissues

**Figure 3:**
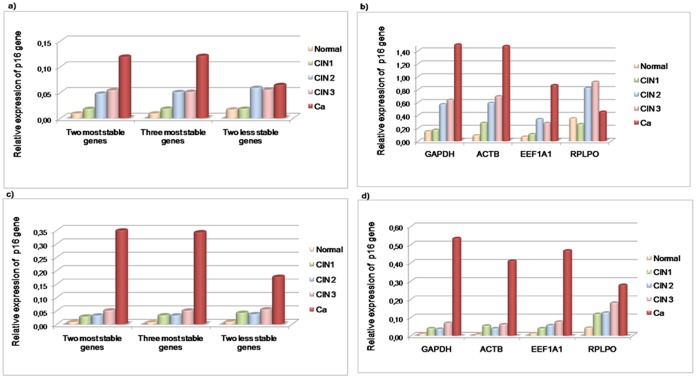
Effect on the p16^INK4a^ expression profile across cervical tissues using different normalizers. In this diagram: the normal tissue is represented by the blue bar; CIN1 is represented by the red bar; CIN2 is represented by the green bar; CIN3 is represented by the pink bar; and cancer (Ca) is represented by the purple bar. The graphs a) and c) show the p16^INK4a^ expression profile by means of the NFs obtained from the individual Ct analysis, as well as the NFs from the pooled Ct analysis, respectively. The graphs b) and d) show p16^INK4a^ expression normalized to individual Ct values and the pooled Ct values of each single candidate reference gene.

For validation purpose, the relative quantification of the p16^INK4a^ target was assessed by using a combination of the two most stable genes, the three most stable genes and the two least stable genes. Target gene expression was normalized through a stability ranking of the genes based on an analysis of individual Ct values (Figure 3a), as well as an analysis involving pooled Ct values (Figure 3c). The overexpression of p16^INK4a^ has been linked to the severity of premalignant lesions, i.e. there is a greater expression of this protein in CIN2 and CIN3 (which corresponds to a high-grade squamous intraepithelial lesions-HSIL) than in CIN1 which corresponds to a low-grade squamous intraepithelial lesions-LSIL [32], [33]. In view of this, p16^INK4a^ expression levels across cervical tissues were reproduced more effectively by normalizations with a combination of the two or three most stable genes (*GAPDH* and *ACTB*; *GAPDH*, *ACTB* and *EEF1A1*) obtained from an analysis of individual Ct values (Figure 3a). Conversely, the p16^INK4a^ profiling based on the normalization factors (NF) from the pooled Ct values varied, and showed similar expression levels between CIN1 (LSIL) and CIN2 (HSIL) (Figure 3c). In addition, as can be seen in Figure 3a and Figure 3c, the use of the two least stable genes resulted in a discrepant expression profile of p16^INK4a^.

The relative quantification of p16^INK4a^ was also assessed; this used a single reference gene that took account of individual Ct values (Figure 3b), as well as pooled Ct values (Figure 3d). In both types of analysis, the results showed that the use of a single reference gene can lead to discrepancies. In Figure 3b, it can be observed that the use of *GAPDH* and *ACTB* resulted in a similar expression pattern to p16^INK4a^ when this is compared with the combination of both genes (*GAPDH* and *ACTB*) from an analysis of individual Ct values (Figure 3a). However, the expression levels of p16^INK4a^ were higher in all instances (Figure 3b and Figure 3d) than the normalization carried out by the combined genes provided by the geNorm (Figure 3a and Figure 3c).

**Figure 4:**
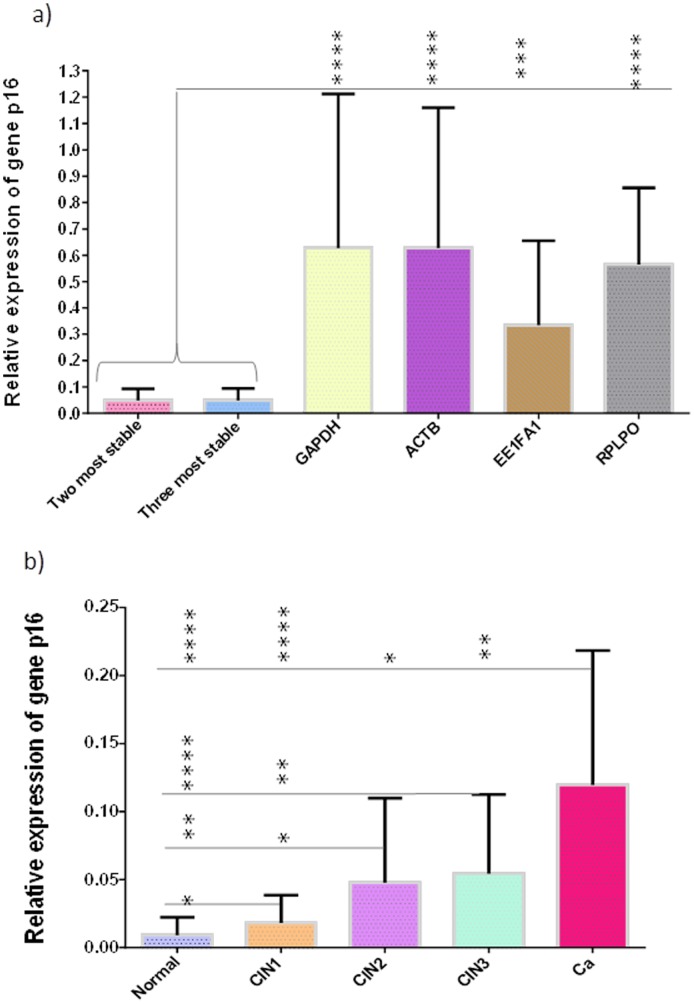
Effect of normalization options on p16^INK4a^ gene expression in cervical tissues. In a), it is shown that there is no significant effect on normalization between the use of the two most stable genes and the three most stable genes. The discrepancies in expression levels of p16^INK4a^ were statistically significant with ANOVA with the use of *GAPDH*, *ACTB*, *EEF1A1* and *RPLPO* as individual normalizers, when compared with the use of the two most stable genes or the three most stable genes. Graph b), shows the expression levels of p16^INK4a^ in cervical tissues using the combination of *GAPDH* and *ACTB* (obtained from the analysis involving individual Ct values) as the normalizer. The error bars indicate a 95% confidence interval; *, p<0.05; **, p<0.01; ***, p<0.0005; ****, p<0.0001.

The relative quantification of the p16^INK4^ transcript in all the cervical tissue conditions was better represented by using the two most stable genes (*GAPDH* and *ACTB*) based on the analysis with individual Ct values (Figure 3a). Thus, in Figure 4a significant differences are shown between this normalization strategy and a strategy involving each single gene, which also takes account of the individual Ct values (Figure 3b). As previously demonstrated, the combination of the two or three most stable genes from the individual Ct values did not change the expression profile of p16^INK4a^. Hence, the target gene expression does not significantly differ if two reference genes are used rather than three (Figure 4a). Conversely, the use of a single reference gene significantly differs when compared to the normalization based on the two and three most stable genes (Figure 4a). In view of this, the combined use of *GAPDH* and *ACTB* for the normalization of the target expression significantly reduced the magnitude of error when compared with the use of a single gene.

Additionally, in Figure 4b the normalized expression levels of p16^INK4a^ are demonstrated through a combination of *GAPDH* and *ACTB* (based on the analysis involving individual Ct values). It should be noted that p16^INK4a^ has shown a significant overexpression in all the cervical tissue conditions, except between CIN2 and CIN3.

### Validation of reference genes for measuring microRNA expression in cervical tissues

**Figure 5:**
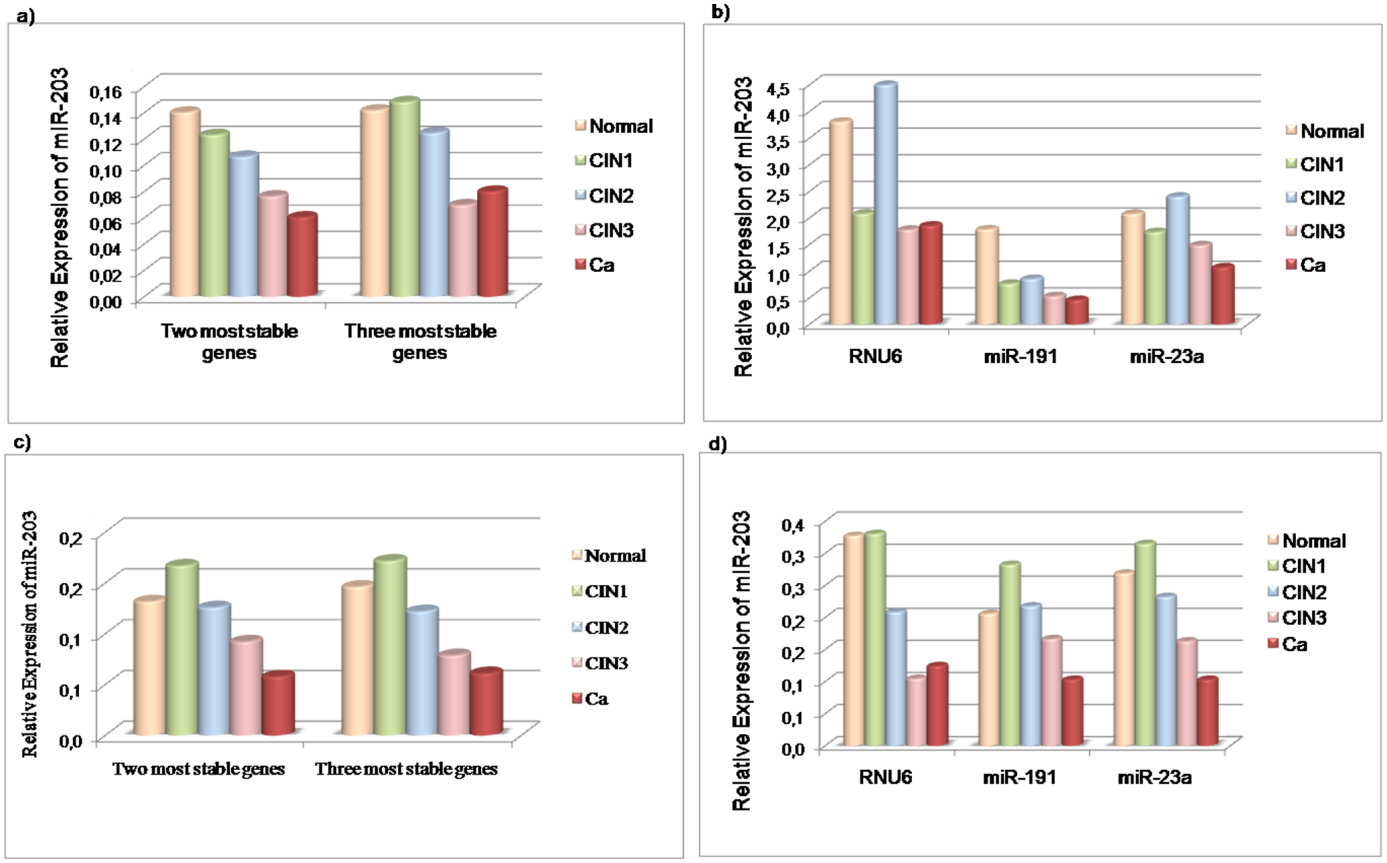
Effects of normalizers on the expression profile of miR-203 across cervical tissues. In this diagram: the normal tissue is represented by the blue bar; CIN1 is represented by the red bar; CIN2 is represented by the green bar; CIN3 is represented by the pink bar; and cancer (Ca) is represented by the purple bar. Graphs a) and c) show miR-203 expression profile using NFs obtained from individual Ct analyses, as well as NFs from pooled Ct analyses, respectively. Graphs b) and d) shows miR-203 expression normalized to individual Ct values and the pooled Ct values of each single candidate reference gene.

The validation of the best normalizers for measuring miRNA expression across cervical tissues was based on a combination of the most stable npcRNA genes obtained by the geNorm analysis, as well as each single gene. The two stability rankings provided by the geNorm (with individual Ct values and pooled Ct values) were identical. However, the use of combined genes (from both analyses) to normalize the relative quantification of miR-203, has reflected variations in its expression profile throughout all the cervical carcinogenesis (Figure 5a and Figure 5c). In a similar way to the results obtained for the measurement of the p16^INK4a^ expression levels; miR-203 demonstrated an expected pattern of ‘downregulated’ expression across cervical tissues [15], [25], [34], [35], when the two most stable genes (*miR-191* and *miR-23a*) were used that originated from the geNorm analysis with individual Ct values (Figure 5a). In Figure 5c a suggested ‘upregulation’ can be observed in the target expression from the normal tissue to CIN1 when the two or three most stable genes are used (based on an analysis involving pooled Ct values). An analysis was also conducted with each candidate gene as a single normalizer using individual Ct values (Figure 5b) as well as pooled Ct values (Figure 5d). Wider discrepancies in the miR-203 expression profile were observed when each candidate gene was used as a single reference. For example, the use of *RNU6* as a normalizer resulted in a discrepant expression profile with elevated expression of miR-203 in normal tissue and CIN2 when individual Ct values were employed (Figure 5b), as well as in normal and CIN1, when pooled Ct values were employed (Figure 5d).

**Figure 6:**
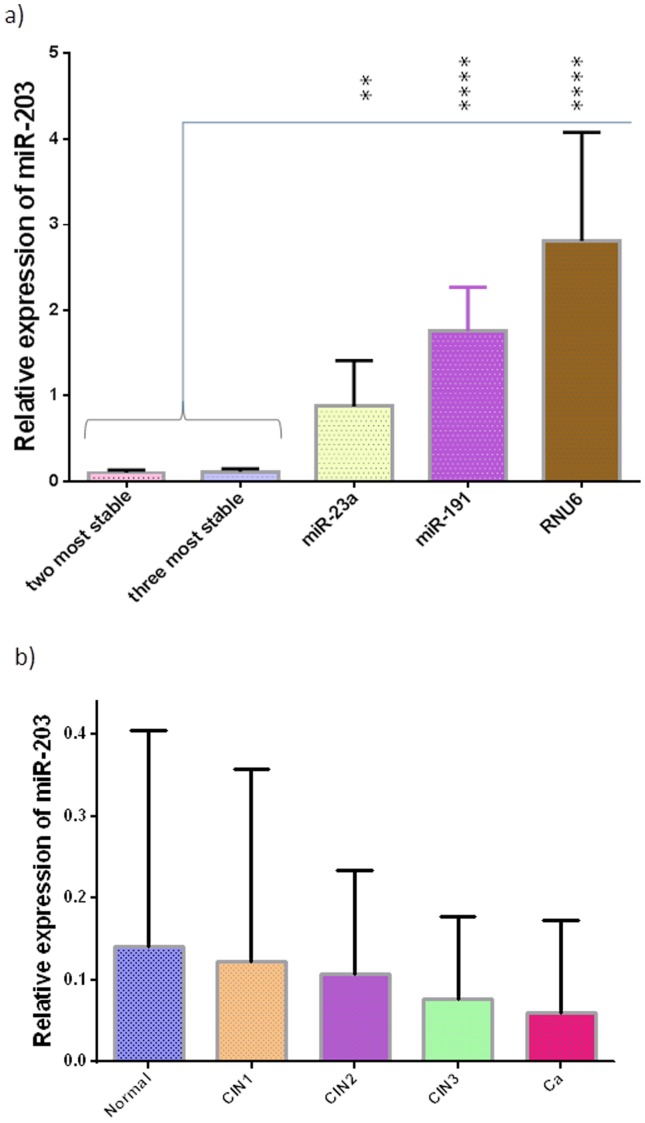
Effect of normalization options on miR-203 expression in cervical tissues. In a), it is shown that there is no significant effect on normalization between the use of the two most stable genes and the three genes. The differences in the expression levels of miR-203 were statistically significant with ANOVA when the use of the two and three most stable genes were compared with the use of each gene as a single normalizer. Graph b), shows the relative quantification of miR-203 in cervical tissues using a combination of *miR-191* and *miR-23a* (from an analysis involving individual Ct values) as the normalizer. The error bars indicate a 95% confidence interval; **, p<0.01; ****, p<0.0001.

Given the factors outlined above, the combination of *miR-191* and *miR-23a* based on the analysis with individual Ct values, was suggested as the best normalizer for measuring miRNA expression in cervical tissues because this provided a better representation of the miR-203 expression profile across cervical carcinogenesis. Nevertheless, no significant difference was observed in the effects on normalization between the use of two or three genes (Figure 6a). However, target gene expression differs significantly when a single reference gene is used (that takes account of individual Ct values) when compared with the normalization obtained by the two or three most stable genes (Figure 6a).

Figure 6b shows normalized expression levels of miR-203 across cervical tissue conditions by the combination of *miR-191* and *miR-23a* obtained from the individual Ct analysis. Interestingly, no significant differences were detected in the expression levels of the target between all the tissues, despite the fact that miR-203 has been reported to be downregulated across cervical carcinogenesis.

## Discussion

Studies of gene expression profile in cervical tissues (normal and neoplastic) have been performed to find biomarkers for cervical cancer [23], [36]. Some of these studies showed discrepant results, e.g. in more recent studies of miRNA expression in cervical carcinogenesis [15], [25], [37]. Some authors suggest that these discrepancies can be attributed to the different platforms and methods employed and the diversity of the samples of the control groups, perhaps due to ethnic variability [15], [37]. However, the use of unsuitable reference genes seems to be one of the reasons for the differences in the results obtained in qPCR studies [16], [38]. The importance of choosing a correct standardization strategy has already been emphasized, both in the qPCR analysis of mRNA [21], and in the miRNA profiles [22].

To the best of our knowledge, the present study is the first to perform a geNorm analysis with a set of samples which meet all of the cervical tissue conditions: Normal + CIN1 + CIN2 + CIN3 + Cancer. This strategy enables reference genes to be used for the identification of potential biomarkers, not only in normal and cancer, but also in premalignant lesions. In view of the examples of failure in the current tests for screening premalignant cervical lesions [3]–[6], these biomarkers could be useful to distinguish between CIN1 and CIN2, CIN2 and CIN3, and cancer as well as in providing more information about the severity and progression of these lesions. Additionally, we have chosen commonly used reference genes for qPCR studies of mRNA [19], [23], [29], [30] and miRNA expression levels in cervical cancer [15], [20], [22], [31] with the aim of validating them in our specific experimental design, as recommended by the MIQE guidelines [10]. The determination of gene expression stabilities was performed with the aid of geNorm software, first developed by Vandesompele, *et al*. in 2002 [12], and since then widely adopted to evaluate the expression stability of the candidate reference genes [39], [40].

In addition, to our knowledge, this is the first time that a study has evaluated the effects of pooling Ct values across replicates (by simulating a pool of samples) on an expression stability analysis, as well as on the qPCR results with regard to cervical tissues. We have proposed to make a comparison between the use of pooled Ct values and individual Ct values (by treating samples as independent) in a qPCR assay, since pooling samples (or RNA) is an alternative method that reduces the costs incurred by a qPCR assay. In this regard, we provide evidence that the use of pooled Ct values is not a strategy to obtain valid qPCR data that is as reliable as a strategy that employs individual Ct values. This evidence was obtained by validating reference genes for measuring mRNA and miRNA expression in cervical tissues. The p16^INK4^ expression profile across all the cervical tissue conditions was represented better by means of the two most stable genes (*GAPDH* and *ACTB*) obtained from the geNorm analysis with the individual Ct values. This strategy has provided an expression profile of p16^INK4a^ that corresponds to that of other studies, which have found a greater expression of p16 protein in HSIL than in LSIL [9], [32], [33]. Similarly, miR-203 displayed a suggestive pattern of downregulated expression [15], [25], [34], [35], with the use of the two most stable genes (*miR-191* and *miR-23a*) derived from the geNorm analysis with the individual Ct values. The use of the two or three most stable genes from an analysis that involve pooled Ct values, is able to cause discrepancies in the expression profiles of both p16^INK4a^ and miR-203. Thus, the results clearly suggest that pooled Ct values can lead to a misinterpretation of the qPCR data. Some studies have included a pool of samples (or RNA pool) in the qPCR assays to reduce biological variability and also to reduce the costs of the experiments [14]–[17]. However, we suggest that the effect of including pooled samples should be evaluated for each experiment which involves qPCR assays, since our study shows that the maintenance of inter-individual variability is a key factor which can ensure the reliability of the qPCR data in cervical tissues.

To date, only two studies have investigated a correct strategy for data normalization in qPCR assays for cervical tissues; one of these studies recommended reference genes for mRNA expression studies [19], and the other for microRNA expression studies [20]. According to Shen *et al*. [19] *EEF1A1* was the most stable gene (followed by *GAPDH* and *RPLP0*) for mRNA quantification in human cervical tissues. Interestingly, in the same work, *ACTB* was found to be the least stable gene in cervical tissues, in contrast with our study where *ACTB* was the second most stable gene. This variation may be linked to the geNorm analysis carried out in our study, which includes all the cervical tissue conditions, as well as the use of different platforms by the laboratories, or else it may be due to ethnic variability. In this way, this data strengthens the need to validate reference genes in a specific experimental setting. With regard to non-protein coding genes, the combined use of *miR-191* and *miR-23a*, based on the analysis of individual Ct values, was the best normalizer for measuring the miRNA target expression in accordance with Shen *et al*. [20]. The use of these two most stable genes reflected the profile of miR-203 across cervical carcinogenesis that was most expected, even though expression levels between cervical tissues have no significance, perhaps due to the small size of the sample [41], [42]. Thus, larger sample sizes are required to obtain valid conclusions.

Even though it has been well established that a normalization strategy is an essential component in ensuring the reliability of the qPCR data, and that reference genes must be validated for each particular experimental setting [10], [11], [21], [22]; a large number of studies of cervical cancer continue to use the most well-known reference genes, on the basis of previous studies and without proper validation, or without mentioning whether this stage has been carried out accurately [23]–[27]. For this reason, in evaluating the effects of using a single reference gene to normalize the target expression, we have assessed the relative expression of p16^INK4^ obtained by each of the four single reference genes (*GAPDH*, *ACTB*, *EEF1A1*, *RPLPO*), and the relative expression of miR-203 obtained by each of the three single reference genes (*RNU6*, *miR-23a*, *miR-191*). The relative expression of both the targets (p16^INK4^ and miR203), which were normalized by each single reference gene, did not show exactly the same pattern as the expression profile obtained by the most suitable normalizer observed in this study: the combination of the two most stable genes provided by the geNorm analysis, involved individual Ct values. The results suggested that the use of a single reference gene can lead to discrepancies in the qPCR data, which is in agreement with the findings of other studies [12], [43], [44]. Apart from this, Shen *et al*. 2010 [19] recommends *EEF1A1* as the most stable gene that can be used as a single reference gene for normalization in gene profiling studies involving human cervical tissues. In our study, the use of *EEF1A1* as a single normalizer led to an erroneous normalization up to 3.0-fold when compared with the normalization where two or three of the reference genes are used together (as shown in Figure 4a). Our findings are in agreement with those of Vandesompele *et al*. [12] where it is stated that “a conventional normalization strategy based on a single housekeeping gene leads to erroneous normalization up to 3.0- and 6.4-fold in 25% and 10% of the cases, respectively, with sporadic cases showing error values above 20”. In this way, it should be stressed that the suitability of reference genes in some studies does not necessarily apply to others. Other authors also recommend the use of at least two reference genes for human tissues [43], [44]. However, even though it has been widely accepted that one of the best ways to normalize the qPCR data is to use at least 2 to 3 reference genes, several studies of cervical cancer continue to use the most well-known reference genes such as *GAPDH* [23], [45], *ACTB* [30], EEF1A1 [46] and *RNU6* [15], [24]–[27], [46]–[51], as a single reference gene and without mentioning whether this stage has been performed accurately. Furthermore, even though it has been established that a normalization standard must reflect the quantity and size of the target of interest to obtain comparable samples [12], [22]; some studies have used an mRNA as a reference gene to normalize the miRNA expression levels in cervical cancer [25], [51]. The use of an unvalidated or single reference gene in qPCR remains a recurring problem that has been addressed and critically discussed in recent papers [38], [40], [52].

## Conclusion

An increasing number of publications have used qPCR to identify differentially expressed messenger RNAs as well as microRNAs between several types of tissues and cells, in various biological conditions or experimental situations. qPCR has become the most widely used technique in these studies due to its simplicity and that fact that it can provide results quickly. However, careful standardization of each stage is of crucial importance to obtain accurate data, such as the inclusion of validated reference genes. In this study, we performed the validation of reference genes for mRNA and miRNA quantification in cervical carcinogenesis. We have made a serious attempt to evaluate the effects of important issues in qPCR assay to ensure accurate data, since the main purpose of our line of research is to identify changes in mRNA and miRNA expression which have a real significance in cervical carcinogenesis. It should be underlined that the suitability of reference genes in some studies does not necessarily apply to others and that the use of a single reference gene is not sufficient to obtain reliable qPCR data; even though several studies continue to employ this methodology in qPCR studies on cancer research. It is worth noting that the best combination of reference genes which can be used for the measurement of targets in this study was selected after a comparison had been made between the use of individual Ct values and pooled Ct values. The results clearly showed that pooled Ct values can lead to an unreliable results, which suggests that studies on cancer research by means of a qPCR assay, should take into account the individuality of each biological sample. Finally, we believe that this study raises important issues and points to the need for further research that is not confined to the area of cervical cancer, but also leads to the question of the qPCR assay.

## Supporting Information

Figure S1 **Real-time PCR standard curve of all primer pairs.** The slope of the standard curves indicates the efficiency of qPCR.
(DOCX)

Figure S2 **Melting peaks of all primer pairs.** The specificity of all the primer pairs was confirmed by a single peak in the melting curve.
(DOC)
