# Supplementary material for: Quantifying mRNA and MicroRNA with qPCR in Cervical Carcinogenesis: A Validation of Reference Genes to Ensure Accurate Data
Source: PLoS One. 2014 Nov 3;9(11):e111021. doi: 10.1371/journal.pone.0111021 (PMC4217744; doi:10.1371/journal.pone.0111021)
Supplement: Figure S1 — Real-time PCR standard curve of all primer pairs. The slope of the standard curves indicates the efficiency of qPCR. (DOCX) [file pone.0111021.s001.docx]

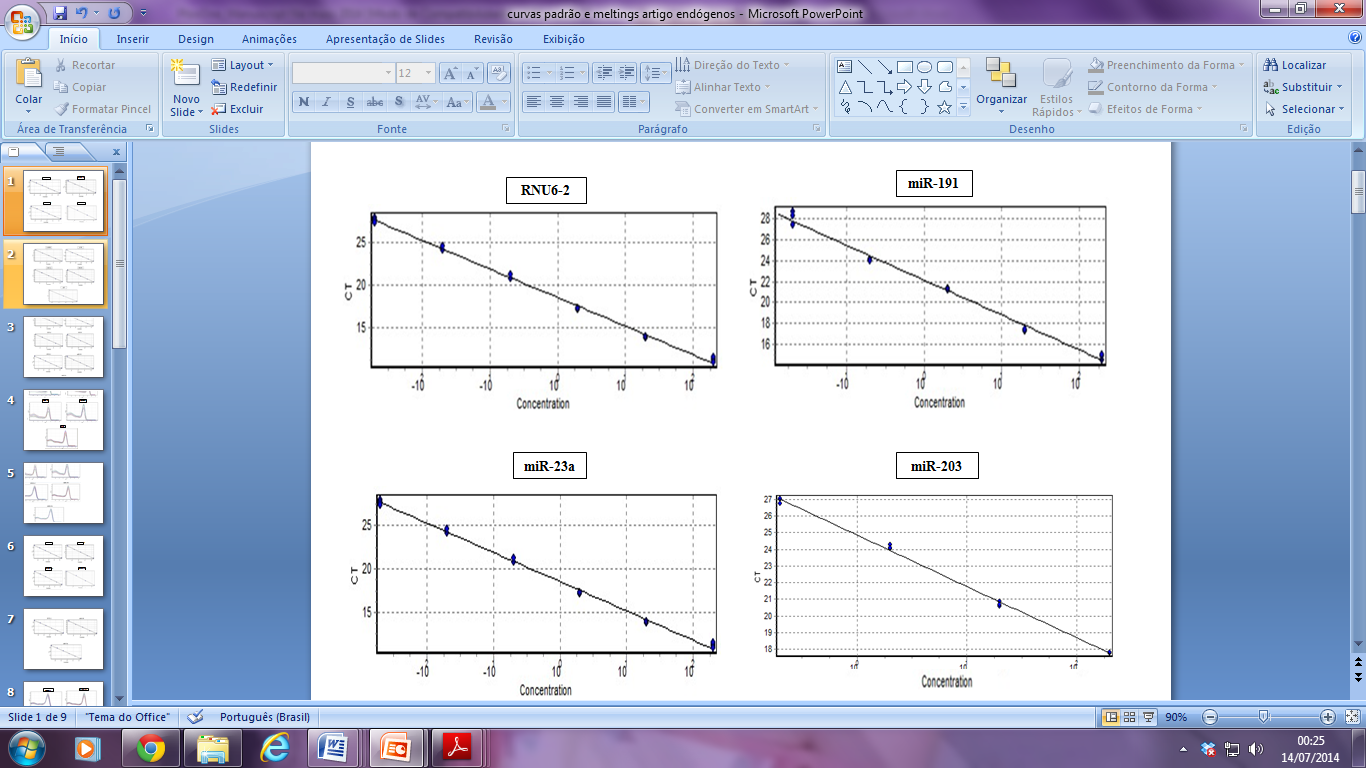

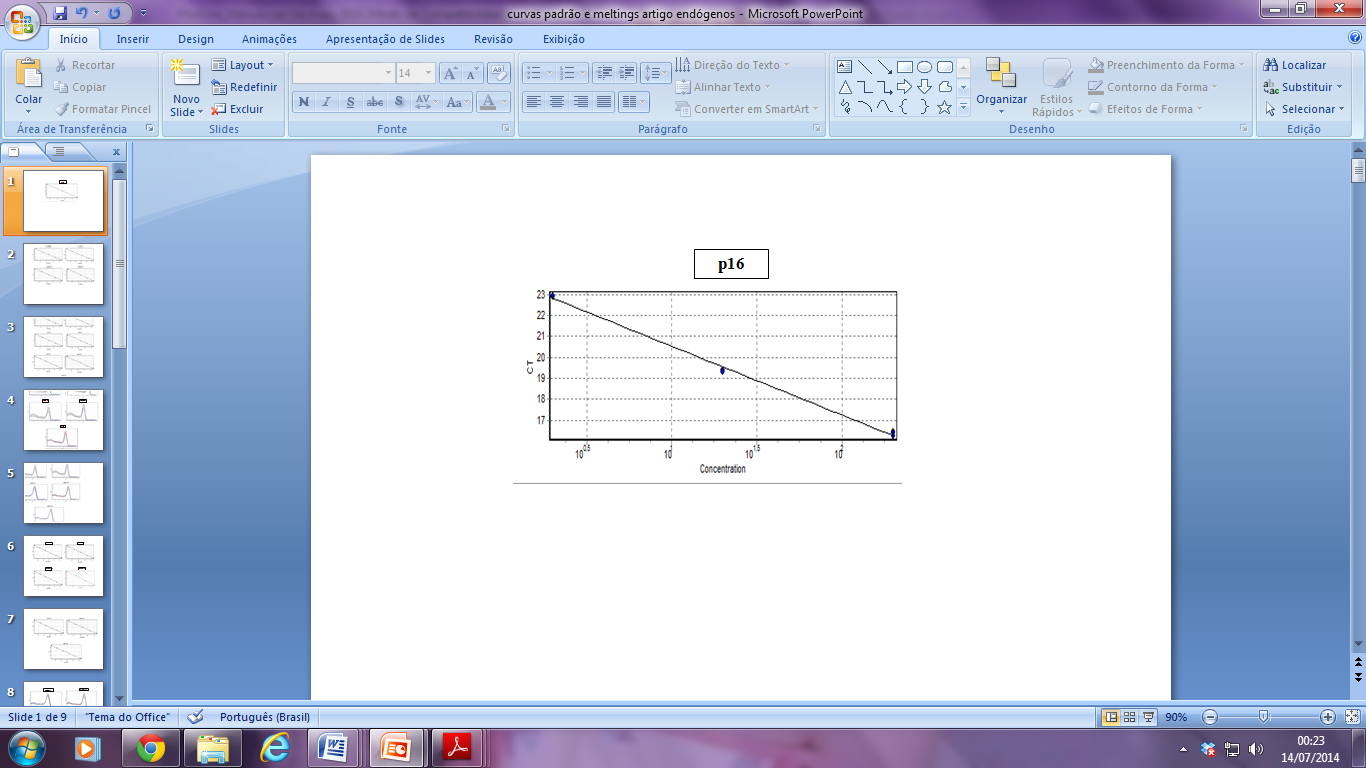

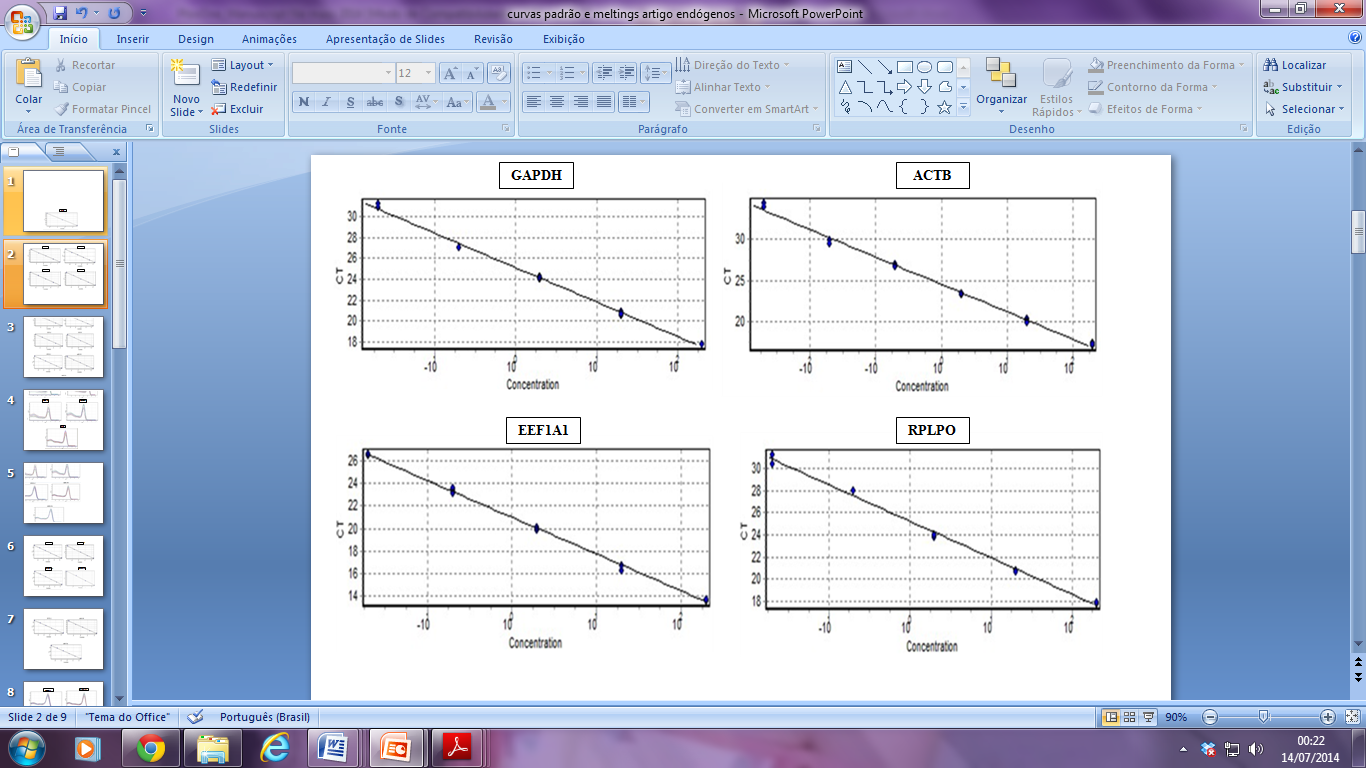


Figure S1. Real-time PCR standard curve of all primer pairs. The slope of the standard curves indicates the efficiency of qPCR.
