# Supplementary material for: Quantifying mRNA and MicroRNA with qPCR in Cervical Carcinogenesis: A Validation of Reference Genes to Ensure Accurate Data
Source: PLoS One. 2014 Nov 3;9(11):e111021. doi: 10.1371/journal.pone.0111021 (PMC4217744; doi:10.1371/journal.pone.0111021)
Supplement: Figure S2 — Melting peaks of all primer pairs. The specificity of all the primer pairs was confirmed by a single peak in the melting curve. (DOC) [file pone.0111021.s002.doc]

**
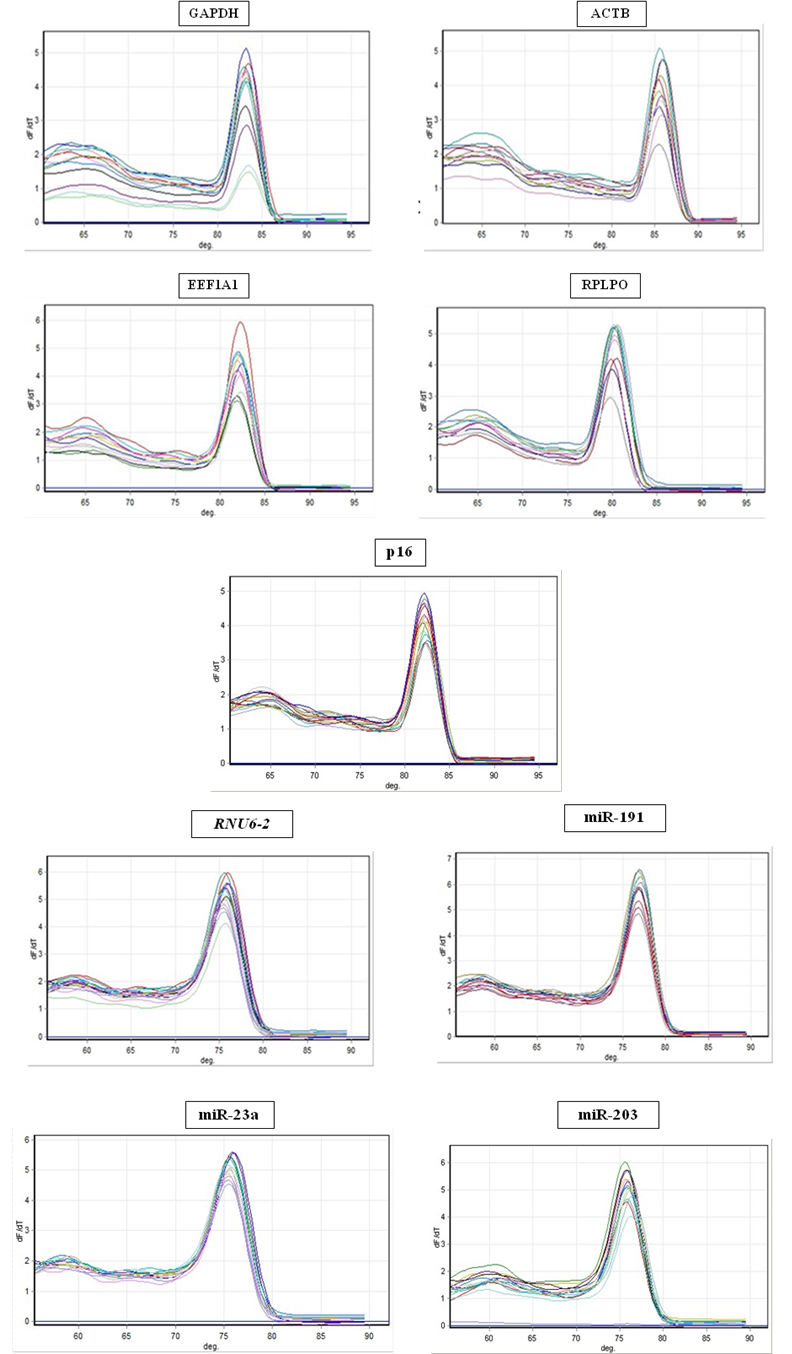
**

Figure S2. Melting peaks of all primer pairs. The specificity of all the primer pairs was confirmed by a single peak in the melting curve
